# Sepsis protects the myocardium and other organs from subsequent ischaemic/reperfusion injury via a MAPK-dependent mechanism

**DOI:** 10.1186/s40635-014-0035-9

**Published:** 2015-01-31

**Authors:** Criona M Walshe, John G Laffey, Leo Kevin, Daniel O’Toole

**Affiliations:** Department of Anaesthesia, Galway University Hospitals and National University of Ireland, University Road, Galway, Ireland

**Keywords:** Sepsis, Preconditioning, Myocardium

## Abstract

**Background:**

Sepsis has been shown to precondition the intact heart against ischaemia/reperfusion (IR) injury, and prior endotoxin exposure of cells in *in vitro* models has shown evidence of protection against subsequent simulated ischaemia. Our aim in this study is to validate these findings and further investigate the signaling pathways involved.

**Methods:**

Adult male Sprague Dawley rats were randomised to control (*n* = 7) or caecal ligation and perforation (CLP)-induced sepsis (*n* = 7). Hearts were harvested at 48 h, suspended in Langendorff mode and subjected to 30-min global ischaemia followed by 90-min reperfusion. In subsequent experiments, designed to determine the mechanisms by which sepsis protected against ischaemic injury, endotoxin-stimulated isolated cardiomyocytes, pulmonary A549 cells and renal HK2 cells were subjected to normoxic and hypoxic conditions. The roles of key pathways, including mitogen-activated protein (MAP) kinases extracellular-regulated protein kinase (ERK) 1/2, p38 MAPK (p38), c-Jun NH2-terminal protein kinase (JNK)), and nuclear factor-kappaB (NF-κB) were examined.

**Results:**

Systemic sepsis protected isolated hearts from subsequent ischaemic/reperfusion-induced injury, enhancing functional recovery on reperfusion [developed left ventricular pressure ((d)LVP) mean(SE) 66.63(±10.7) mmHg vs. 54.13(±9.9) mmHg; LVP_max_ at 60 min 67.29(±11.9) vs. 72.48(±9.3), sepsis vs. control] despite significantly reduced baseline LV function in CLP animals (*p* < 0.001). Septic preconditioning significantly reduced infarct size after IR injury (*p* < 0.05). Endotoxin exposure protected isolated cardiomyocytes against hypoxia-induced cell death (*p* < 0.001). This effect appeared mediated in part via the p38, JNK and NF-κB pathways, but was independent of the ERK pathway, and did not appear to be mediated via HMGB1. The preconditioning effect of endotoxin was also demonstrated in isolated kidney and lung cells, suggesting that this preconditioning effect of sepsis is not confined to the myocardium.

**Conclusions:**

Sepsis preconditions the isolated rat heart against myocardial IR injury. These effects appeared to be mediated in part via the p38, JNK and NF-κB and pathways, but were independent of the ERK and HMGB pathways.

## Background

Sepsis remains a major cause of mortality, and there is recent data to suggest that the incidence of sepsis is increasing [1]. Treatment options remain limited to early antibiotic therapy, resuscitation and aggressive support of failing organ systems. Multiorgan dysfunction syndrome (MODS), the most severe end of the spectrum of sepsis syndrome, may involve the myocardium [2,3], and this has clear prognostic implications, with some reports pointing to a direct correlation between the onset of myocardial dysfunction and mortality in septic shock [4].

The pathophysiology of septic cardiomyopathy has not been fully elucidated. Although apoptosis is postulated to play an important role in the pathophysiology of septic cardiomyopathy [5,6], *post*-*mortem* studies have demonstrated relatively little cell death, even in cases of undoubted severe septic cardiomyopathy [7,8]. This has led to the suggestion that activation of pro-survival pathways may occur in association with pro-apoptotic pathway activation and that septic cardiomyopathy, although ultimately dysfunctional for the organism, is a reactive process of metabolic downregulation, serving a cellular preservation function broadly similar to preconditioning [6].

Preconditioning is the phenomenon whereby prior exposure to a stimulus, often one normally held to be injurious, triggers phenotypic changes that confer resistance to subsequent insults. It was first described by Murry et al. in 1986 [9], following the discovery that brief episodes of non-lethal myocardial ischaemia, followed by brief periods of reperfusion, provided protection against a subsequent more prolonged period of ischaemia/reperfusion. The concept of endotoxin-induced increased tissue tolerance first emerged in the 1960s [10]. These early descriptions of ‘endotoxin tolerance’ in animal models were followed by discoveries that a similar phenomenon occurs in humans [11]. Sepsis preconditioning of cardiac tissue may also occur in response to brief, non-fatal episodes of sepsis and bears undoubted observational similarities to ischaemic preconditioning, being inhibited, for example, by cycloheximide, a well-known blocker of ‘*late ischaemic preconditioning*’ [12]. The mechanisms whereby sepsis confers protection to the heart against subsequent ischaemia/reperfusion injury, however, are unknown, and whether this phenomenon most closely approximates myocardial stunning, preconditioning or other unrelated effects remains to be firmly established.

In the current study, we hypothesised that sepsis would protect the myocardium from subsequent ischaemia/reperfusion (IR) injury. We further hypothesised that the mitogen-activated protein kinases (MAPK) and nuclear factor-kappaB (NF-κB) pathways mediated this protective effect.

## Methods

### *Ex vivo* isolated heart model

All animal studies were carried out with the approval of the National University of Ireland Galway Animal Care Research Ethics Committee and under licence issued by the Public Health Division, Department of Health, Ireland. Specific pathogen-free adult male Sprague Dawley rats (Charles River Laboratories, Kent, UK), 250 to 300 g in body weight, were used for caecal ligation and perforation (CLP)-isolated heart experiments. Animals were randomised to control (*n* = 7) or CLP (*n* = 7) groups. The CLP procedure was carried out under general anaesthesia. Briefly, following midline laparotomy, the caecum was identified and ligated, following which needle perforation was carried out, and wound layers were closed. Animals were returned to housing in individually ventilated cages. At 48 h following operation, animals were harvested for isolated heart protocol using the Langendorff suspension. Animals were sacrificed by means of decapitation. The heart was then removed and suspended from a canula inserted into the aortic root facilitating perfusion with prewarmed Krebs Ringer at 37°C, oxygenated by means of a gas mixture containing 5% CO_2_ and 95% O_2_, maintaining pH of solution at 7.4. Episodes of ventricular fibrillation were promptly treated with lignocaine bolus 200 mg, administered via a t-piece connected to the aortic canula such that it passes directly through the coronary arteries. Measurements of systolic and diastolic left ventricular (LV) functional characteristics were made with a saline-filled balloon placed in the left ventricle. This balloon was connected to a pressure transducer facilitating provision of a digital readout as a continuous waveform. Balloon volume was adjusted to achieve LVEDP 4 to 8 mmHg, following which the volume of the balloon was maintained constant, thus providing a pre-load-independent measure of contractility. Parameters measured were systolic and diastolic left ventricular pressure (LVP), developed (sys-dias) LVP, maximal and minimal derivatives of LVP (dp/dt LVP_max_, dp/dt LVP_min_) and heart rate. Global myocardial ischaemia was induced by means of clamping the inflow line for a period of 30 min, followed by 90 min reperfusion. Upon completion of the protocol, the hearts were stained with 1% 2,3,5-triphenyltetrazolium chloride (TTC), a dye taken up only by viable cells. Myocardial infarct size was determined by direct visualisation, dissection and separation of viable and infarcted heart tissue, which were then weighed and expressed as percentage of total heart weight.

### *In vitro* hypoxic/ischaemic studies

Preparation of primary cultures of neonatal cardiomyocytes was achieved by trypsin/collagenase digestion of ventricles harvested from 3- to 5-day-old neonatal Sprague Dawley rats following decapitation. Cells were cultured in a 1:1 mixture of Dulbecco's modified Eagle's medium (DMEM) and Ham's F12 (D8437 Sigma-Aldrich Ireland Limited, Wicklow, Ireland) containing 17 mM glucose, supplemented with 1 mM sodium pyruvate (Gibco, Life Technologies, Grand Island, NY, USA), 10% newborn foetal calf serum, 5 μg/ml bovine insulin, 5 ng/ml human transferrin, 5 μg/ml sodium selenite, 100 U/ml penicillin and 100 μg/ml streptomycin. All plates and flasks used to culture cardiomyocytes were previously coated with 2% gelatin solution for 24 h and aspirated prior to plating of cells, to ensure adherence of cardiomyocytes. To prevent fibroblast proliferation, 100 mM 5-bromo-2-deoyuridine (BrdU) was added to the cardiomyocyte media for 48 h following plating, at which point media were replaced. Each well was then re-fed with prewarmed media prior to commencing experimentation. This is required to provide sufficient metabolic substrate for viability and normal function throughout the experimental period.

Human alveolar epithelial cells (A549) were purchased from ATCC, Middlesex, UK. HK2 human adult kidney cells were a kind gift from Prof. Michael Ryan (University College Dublin, Ireland). Cells were cultured in DMEM medium supplemented with heat-inactivated 10% foetal bovine serum, 2 mmol/l glutamine, 100 U/ml penicillin and 100 μg/ml streptomycin.

To investigate putative signaling pathways involved in the protective effect of cellular exposure to endotoxin prior to incubation under hypoxic conditions, inhibitors of MAP kinases extracellular-regulated protein kinase (ERK) 1/2, p38 MAPK (p38), and c-Jun NH2-terminal protein kinase (JNK) in addition to pyrrolidinedithiocarbamate (PDTC) (an NF-κB inhibitor) and anti-HMGB1 antibody were added to cell culture at standard concentrations.

For cell viability experiments, A549 and HK2 cells were grown to confluence in 96-well flat-bottom tissue culture plates (Sarstedt, Nümbrecht, Germany) and cultured in pre-equilibrated and prewarmed RPMI, DMEM and a 1:1 mixture of DMEM:Ham's F12 media, respectively. The cells were allowed 24 h to polarise prior to experimentation. The medium was then re-fed, supplemented with LPS, LPS with MAPK inhibitors, PDTC, anti-HMGB1 antibody or vehicle under conditions of normoxia (humidified 5% CO_2_, 95% air) or hypoxia (humidified O_2_/N_2_/CO_2_ in the following ratio: 2:93:5), following randomisation.

Viability was measured by cellular 3-(4,5-dimethylthiazol-2-yl)-2,5-diphenyl tetrazolium bromide (MTT) assay, under conditions of normoxia (humidified 95% air, 5% CO_2_) and hypoxia (humidified O_2_/N_2_/CO_2_ in the following ratio: 2:93:5), for an experimental duration of 48 h, on all four cell types. The MTT assay provides an estimate of mitochondrial activity from which cell viability is deduced.

### Data analysis

Results are expressed as mean (SD) for normally distributed data. Data were analysed using *t* test comparison. A *p* value of 0.05 was considered statistically significant.

## Results

### Sepsis preconditioning protects the heart

Seven animals underwent the CLP procedure to provide a model of polymicrobial sepsis. These septic animals were then sacrificed and hearts suspended in Langendorff preparation. The hearts were also isolated from seven non-septic animals which provided the control group in this series of experiments.

#### Indices of myocardial function

**Figure 1:**
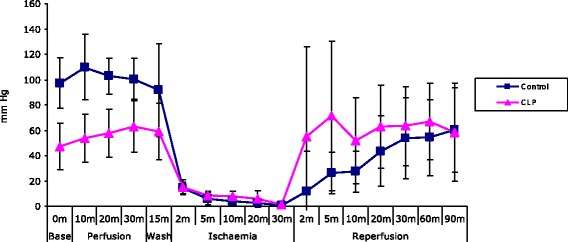
**Sepsis protects the isolated heart against ischaemia/reperfusion injury - (d)LVP.** Line graph depicting developed LVP in isolated hearts, control and animals subjected to the CLP procedure (*n* = 7 per group), demonstrating that sepsis reduces myocardial function with lower (d)LVP at baseline, but protects the heart against ischaemia/reperfusion injury with more rapid functional recovery at reperfusion.

Hearts from septic animals had reduced baseline developed LVP ((d)LVP) compared to those from control animals, mean (SE) 101.11 ± 6.06 mmHg vs. 47.15 ± 6.88 mmHg, *p* = 0.0001 (Figure 1).

**Figure 2:**
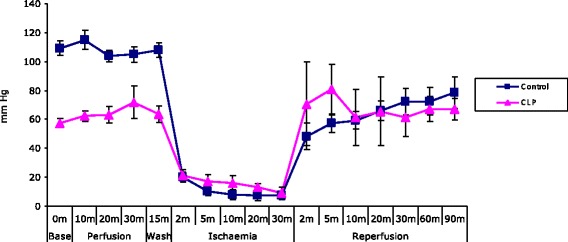
**Sepsis protects the isolated heart against ischaemia/reperfusion injury - LVP**_**max**_ **.** Line graph depicting LVP_max_ in isolated hearts, control and animals subjected to the CLP procedure (*n* = 7 per group), demonstrating that sepsis reduces myocardial function with lower LVP_max_ at baseline, but protects the heart against ischaemia/reperfusion injury with more rapid functional recovery at reperfusion.

LVP_max_ was reduced in CLP animals compared to controls at baseline, 109.08 ± 4.65 mmHg vs. 57.49 ± 7.22 mmHg, *p* = 0.00003 (Figure 2).

During the early reperfusion phase, (d)LVP was greater for CLP hearts than for control, pointing to more rapid functional recovery at reperfusion. This effect was not lasting, however, as at 90-min reperfusion, (d)LVP was similar for CLP and control animals, 58.51 ± 14.59 mmHg vs. 54.27 ± 5.75 mmHg, as was LVP_max_, 67.16 ± 11.05 mmHg vs. 78.41 ± 10.49 mmHg, *p* = 0.77.

Episodes of fibrillation upon reperfusion occurred more frequently in the control group (2/7, 28%) compared to the sepsis group (1/7, 14%). Episodes were promptly terminated by administration of lignocaine.

#### Infarct size

**Figure 3:**
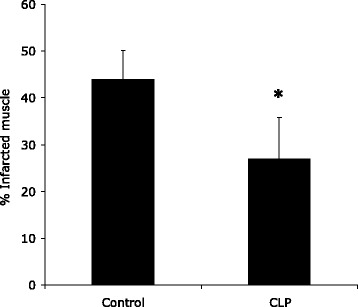
**Sepsis protects the isolated heart against ischaemia/reperfusion injury - infarct size.** Bar chart depicting infarct size in isolated hearts, control and animals subjected to CLP procedure (*n* = 7 per group), demonstrating that sepsis reduces myocardial function with reduced infarct size in the sepsis group. *Significantly different (*p* < 0.05, Students *t* test).

Infarct sizes after ischaemia/reperfusion were significantly reduced in CLP hearts compared to controls (27% ± 8.8 vs. 44% ± 6.2, *p* < 0.05) (Figure 3).

### Mechanism of action of septic preconditioning

#### Cardiomyocytes

**Figure 4:**
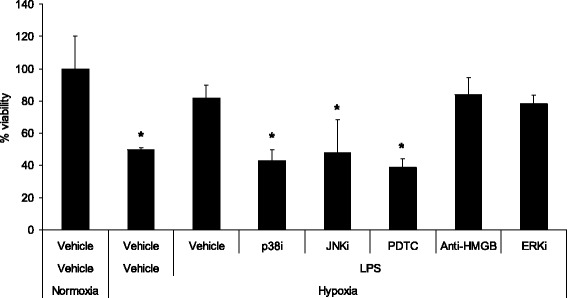
**Endotoxin exposure protects isolated cardiomyocytes from hypoxia-induced cell death.** Bar chart depicting cell viability as measured by MTT assay of cardiomyocytes expressed as % of normoxia controls. Groups were normoxia and hypoxia controls and cells pretreated with LPS and inhibitors prior to incubation under conditions of hypoxia (*n* = 6 per group). The figure demonstrates the protective effect of endotoxin exposure against hypoxia-induced cell death, an effect reversed by exposure to inhibitors of p38 and JNK MAPK and NF-κB inhibition. *Significantly different (*p* < 0.02, Students *t* test).

Endotoxin preconditioning protected against hypoxia-induced cardiomyocyte cell death vs. hypoxic controls [cell viability 81.55% ± 8.2% vs. 49.74% ± 1.1%, respectively, *p* = 0.000125] (Figure 4). Prior blockade of p38, JNK and NF-κB pathways all caused significant reversal of endotoxin preconditioning with viabilities decreased to 43.13% ± 6.6%, *p* = 0.000004, 47.83% ± 20.2%, *p* = 0.01, 38.67% ± 5.6%, *p* = 0.000001, respectively (Figure 4). Addition of inhibitors of ERK 1/2 and anti-HMGB1 antibody demonstrated no significant effects.

#### Alveolar epithelial cells

**Figure 5:**
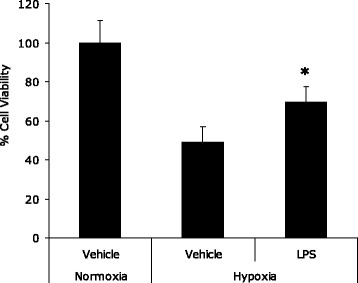
**Endotoxin exposure protects pulmonary alveolar cells from hypoxia-induced cell death.** Bar chart depicting cell viability as measured by MTT assay of A549 cells expressed as % of normoxia controls. Groups were normoxia and hypoxia controls and cells pretreated with LPS prior to incubation under conditions of hypoxia (*n* = 5 per group). The figure demonstrates the protective effect of endotoxin exposure against hypoxia-induced cell death. *Significantly different (*p* < 0.01, Students *t* test).

Endotoxin preconditioning was protective against hypoxia-induced cell death, with viability of 69.7% ± 7.62% vs. hypoxic controls 49.01% ± 7.95%, *p* = 0.003 (Figure 5).

#### Kidney cells

**Figure 6:**
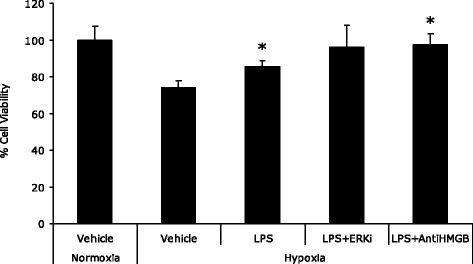
**Endotoxin exposure protects human kidney cells from hypoxia-induced cell death.** Bar chart depicting cell viability as measured by MTT assay of HK2 cells expressed as % of normoxia controls. Groups were normoxia and hypoxia controls and cells pretreated with LPS prior to incubation under conditions of hypoxia (*n* = 6 per group). The figure demonstrates the protective effect of endotoxin exposure against hypoxia-induced cell death. *Significantly different (*p* < 0.02, Students *t* test).

Endotoxin preconditioning was protective against hypoxia-induced cell death, with viability of 85.51% ± 3.27% vs. 73.88% ± 4.04%, *p* = 0.0004 (Figure 6). Addition of anti-HMGB1 antibody and ERK 1/2 inhibitors to LPS-treated cells did not reverse these protective effects, with viabilities of 97.3% ± 6.24% and 96.15% ± 11.7%, respectively, *p* = 0.002 and *p* = 0.007 (Figure 6).

## Discussion

Previous investigators have reported that exposure of hearts to sublethal dosing of endotoxin confers protective effects against myocardial ischaemia/reperfusion injury in a variety of experimental models [13-15], including the Langendorff mode [16-19]. However, data in the literature examining the effects of polymicrobial sepsis, such as that induced by caecal ligation and operation, is less frequently encountered. It should be noted that inherent difficulties exist with experimental models utilising exposure of animal species to endotoxin. Although a reproducible experimental model that is easy to establish, endotoxaemia is not a perfect model of sepsis, with differing cytokine kinetic responses, duration of sepsis and time to death [20]. Making a conceptual advance on LPS exposure as a model of sepsis, we utilised the CLP procedure to induce polymicrobial sepsis. CLP is an *in vivo* model of sepsis that has been shown to be valid and reproducible [21] and is a more accurate replication of systemic inflammatory response to an infectious stimulus [22], more loosely mimicking an important clinically encountered scenario and is considered by some to be an essential preclinical model for sepsis studies [23].

The results of our experiments demonstrate that septic animals sustained marked sepsis-induced myocardial dysfunction, as evidenced by significantly reduced baseline (d)LVP and LVP_max_ compared to control animals, *p* = 0.0001 and *p* = 0.00003, respectively (Figures 1 and 2). Following ischaemia and reperfusion, CLP hearts showed more rapid recovery of functional measures, despite significantly reduced baseline function, and infarct size was significantly reduced in septic hearts (Figure 3). These results strongly support a preconditioning effect of polymicrobial sepsis.

Episodes of fibrillation were seen more frequently in the control group compared to the sepsis group (28% vs. 14%). This likely reflects the severity of IR injury, with protection conveyed in the CLP group conveyed by sepsis preconditioning. The use of lignocaine to prevent or treat reperfusion arrhythmias in the Langendorff model of the isolated heart has been well described [16]. Episodes of fibrillation were promptly treated and therefore unlikely to affect functional outcomes or infarct size.

Studies have shown sepsis preconditioning to demonstrate putative similarities to ischaemic preconditioning, particularly so-called ‘*late preconditioning*’ [12,24]. For example, with induction of gram-negative sepsis, myocardial protective effects are demonstrable at 6 h [18,25], and the myocardial protective effects of LPS exposure were reported to appear after 12 to 24 h [12]. As mentioned previously, the administration of the drug cycloheximide was found to inhibit protection, an effect demonstrable with late ischaemic preconditioning [26]. Additionally, there is strong evidence from multiple sources that the protective effects of LPS utilise signaling pathways known to be of central importance in ischaemic preconditioning, namely NOS2 and Akt [24]. Despite these similarities and intense research in the area of ischaemic preconditioning, the signaling pathway through which sepsis protects the heart from IR injury remains to be fully elucidated.

To investigate the *in vitro* effects of endotoxin exposure, we examined cell viability as measured by the MTT assay. The MTT assay provides an estimate of mitochondrial activity from which cell viability is deduced. In the presence of functioning mitochondria, MTT is reduced from a colourless compound to a blue product (MTT-formazan). The assay involves spectrophotometrical measurement of the concentration of the blue product. This is then calibrated *post hoc* to provide an indirect measure of mitochondrial function.

Lipopolysaccharide exerts its effects through TLR4-dependent NF-κB activation, leading ultimately to pro-inflammatory cytokine release. LPS is known to induce both pro-apoptotic and anti-apoptotic pathways, inducing apoptosis in endothelial cells [26] and hepatocytes [27] while promoting survival in monocytes [28], neutrophils [29], macrophages [30] and cardiomyocytes [4,12]. Although there is evidence to support a protective role played by LPS against myocardial ischaemia and reperfusion injury [13,16,19], less is known about the effects of LPS on apoptosis of cardiomyocytes. The current study in which LPS pretreatment of cardiomyocytes conferred protection against hypoxia-induced cell death (Figure 4) supports the findings of Chao et al. [4] who found reduced apoptosis in cardiomyocytes treated with LPS and subjected to hypoxia and serum deprivation, supporting a preconditioning effect of LPS in *in vitro* models of hypoxia. Limited studies exist outlining the effect of endotoxin preconditioning in other organ systems, and our findings in the current study speak to the fact that these findings can be generalised to other tissues and are not specific to cardiomyocytes through our demonstration of endotoxin preconditioning in alveolar epithelial (Figure 5) and human kidney (Figure 6) cells.

Due to the known similarities between ischaemic and sepsis preconditioning, and the important roles that stress kinases and NF-κB activation are known to play in the signaling of ischaemic preconditioning [31,32], we utilised blockers of the stress kinases, and ammonium PDTC, a direct inhibitor of the NF-κB pathway, to further examine potential signaling pathways involved in sepsis preconditioning.

The MAPK are a family of kinases that have been shown to be activated following multiple extracellular stimuli [33,34], with effects controlling cellular growth and differentiation [35-37]. Three MAPK have been identified in the cardiomyocyte: ERK, p38 and JNK. These are known to play key roles in cellular signal transmission between the cell surface and nuclei [38]. Although the signaling pathways through which sepsis confers myocardial protective effects have as yet to be elucidated, an important role has been proposed for the MAPK JNK, with additional reports of increased TNF-α-induced apoptosis associated with JNK inhibition [39]. This is supported by the findings of the current study, which demonstrates that the addition of JNKi to cardiomyocytes incubated under hypoxic conditions attenuated the protective effects against cell death associated with LPS (Figure 4). p38 MAPK is undoubtedly activated by LPS, although studies have revealed the conflicting effects of attenuated [39] and enhanced [40] apoptosis consequent to this. The results of the current study support a role of increased cell survival, as evidenced by attenuation of LPS-induced protective effects against hypoxia in cardiomyocytes (Figure 4), a finding that warrants further investigation. Although a role has been suggested by ERK 1/2 MAPK in the sepsis preconditioning signaling pathway [4], incubation of cardiomyocytes with inhibitors of ERK 1/2 had no demonstrable effect on endotoxin protection.

Studies have demonstrated an important role of NF-κB activation in delayed ischaemic preconditioning *in vivo* [41], and there is some evidence of a role played in signaling pathways of sepsis preconditioning [42]. In the current study, inhibition of NF-κB through the use of cellular incubation with PDTC attenuated endotoxin preconditioning, providing support to our hypothesis that this mediator may play an important role in sepsis preconditioning.

HMGB1, a so-called endogenous danger signaling molecule, is actively released by activated monocytes and macrophages and is a key late mediator of the inflammatory response to sepsis [43-45], with levels corresponding to the onset of mortality [43,46]. There is evidence that HMGB1 may also have preconditioning effects [47], and for this reason, we utilised anti-HMGB1 antibodies to ascertain whether HMGB1 may play a role in the signaling pathway of endotoxin preconditioning. Our results determined no demonstrable effects with HMGB1 inhibition.

## Conclusions

These studies provide further confirmation of a preconditioning effect of sepsis in the intact heart subjected to global ischaemia/reperfusion. In addition, we have provided evidence of endotoxin preconditioning in cultured cardiomycocytes and further expanded on this to other organ systems through demonstration of effects in pulmonary alveolar cells and human kidney cells. Our investigation of the signaling pathway involved in these effects were reversed by NF-κB, p38 and JNK inhibition, highlighting potential important roles for these mediators in sepsis preconditioning. Taken together, these findings suggest a phenomenon with a high degree of relative organ specificity and requiring a complex signaling pathway.
